# Driving Influences of the Doppler Flash Observed by SuperDARN HF Radars in Response to Solar Flares

**DOI:** 10.1029/2022JA030342

**Published:** 2022-06-03

**Authors:** S. Chakraborty, L. Qian, J. B. H. Baker, J. M. Ruohoniemi, K. Kuyeng, J. M. Mclnerney

**Affiliations:** ^1^ Bradley Department of Electrical and Computer Engineering Virginia Tech Blacksburg VA USA; ^2^ National Center for Atmospheric Research Boulder CO USA; ^3^ Radio Observatorio de Jicamarca Instituto Geofisico del Peru Lima Peru

**Keywords:** space weather, shortwave fadeout, Doppler flash, solar flare, magnetic crochet, SuperDARN

## Abstract

Sudden enhancement in high‐frequency absorption is a well‐known impact of solar flare‐driven Short‐Wave Fadeout (SWF). Less understood, is a perturbation of the radio wave frequency as it traverses the ionosphere in the early stages of SWF, also known as the Doppler flash. Investigations have suggested two possible sources that might contribute to it’s manifestation: first, enhancements of plasma density in the D‐and lower E‐regions; second, the lowering of the F‐region reflection point. Our recent work investigated a solar flare event using first principles modeling and Super Dual Auroral Radar Network (SuperDARN) HF radar observations and found that change in the F‐region refractive index is the primary driver of the Doppler flash. This study analyzes multiple solar flare events observed across different SuperDARN HF radars to determine how flare characteristics, properties of the traveling radio wave, and geophysical conditions impact the Doppler flash. In addition, we use incoherent scatter radar data and first‐principles modeling to investigate physical mechanisms that drive the lowering of the F‐region reflection points. We found, (a) on average, the change in E‐ and F‐region refractive index is the primary driver of the Doppler flash, (b) solar zenith angle, ray’s elevation angle, operating frequency, and location of the solar flare on the solar disk can alter the ionospheric regions of maximum contribution to the Doppler flash, (c) increased ionospheric Hall and Pedersen conductance causes a reduction of the daytime eastward electric field, and consequently reduces the vertical ion‐drift in the lower and middle latitude ionosphere, which results in lowering of the F‐region ray reflection point.

## Key Points

- Changes in the E‐ and F‐region refractive index are the drivers of the Doppler flash
- Characteristics of the Doppler flash are affected by zenith angle, frequency, elevation angle, and flare location on the solar disk
- Lowering of the F‐region ray reflection height is driven by the flare‐enhanced E‐region Hall and Pedersen conductance

## 1 Introduction

A solar flare is a sudden enhancement in solar electromagnetic radiation, specifically in X‐ray and EUV wavebands, causing a brightening of the Sun’s atmosphere (Davies, 1990; Fletcher et al., 2011; Mitra, 1974). Flare‐enhanced electromagnetic radiation reaches the Earth within 8 min and causes an increase in electron density in the dayside ionosphere (Belrose & Cetiner, 1962; Davies, 1990; Gopika et al., 2021; Qian & Woods, 2021; Qian et al., 2011). Sudden enhancement in ionospheric electron density, referred to as a sudden ionospheric disturbance (SID) (e.g., Davies, 1990; Dellinger, 1937), following a solar flare severely disrupts trans‐ionospheric high frequency (HF: 3–30 MHz) communications by introducing HF absorption, and signal anomalies. HF absorption, commonly referred to as short‐wave fadeout (SWF) (e.g., Chakraborty et al., 2018, 2019; Chakraborty, Baker, et al., 2021; Chakraborty, Baker, & Ruohoniemi, 2021; Chakraborty, Ruohoniemi, et al., 2021; Davies, 1990; Fiori et al., 2018, 2022), is a well‐studied phenomenon caused by dissipation of signal energy as heat through collisions with neutral particles, leading to a partial or complete reduction in the strength of the radio signal (e.g., Browne et al., 1995). In contrast, frequency and phase anomalies, commonly referred to as sudden frequency and phase deviation (SFD, SPD), are less explored and understood phenomena (Khan et al., 2005; Kikuchi et al., 1986; Liu et al., 1996; Watanabe & Nishitani, 2013). This study focuses on the statistical characterization of SFDs following solar flares using HF radar (Doppler radar) observations and comparison with simulations from a first principles‐based modeling framework.

A typical signature of SFD in HF radar observations is a sudden rise in the apparent Doppler velocity of the returned signal, also referred to as the “Doppler flash” (Chakraborty et al., 2018). Studies show that Doppler flash is the earliest signature recorded by a Doppler radar following a solar flare and typically lasts for only a few minutes during the initial phase of the SID, while disruption in trans‐ionospheric HF propagation due to flare‐driven radio blackouts lasts for 30–60 min. Comprehensive characterization of the Doppler flash phenomena can provide new insights into the spatiotemporal evolution of solar flare effects (SFEs) generally and unveil information about middle and low latitude ionospheric electrodynamics (e.g., Curto et al., 2018; Sumod & Pant, 2019).

Early studies of SFDs involved analysis of very low frequency (VLF) data (e.g., Khan et al., 2005), ionosondes (e.g., Ellison, 1953), and incoherent scatter radar (ISR) (e.g., Mendillo & Evans, 1974; Pedatella et al., 2018). More recently, Watanabe and Nishitani (2013) used Super Dual Auroral Radar Network (SuperDARN) observations to study the evolution of the Doppler flash. Some studies used data from magnetometers to study the solar flare impact on Sq currents (magnetic crochet), the equatorial electrojet, and other anomalies in electrodynamics (e.g., Alken & Maus, 2010; Curto et al., 2018). Incoherent scatter radar data provides information about intrinsic ionospheric properties such as plasma density, temperature, and electric field. On the other hand, the VLF receivers and SuperDARN HF radars provide insight into ionospheric propagation conditions. The study by Watanabe and Nishitani (2013) provided the first statistical characterization of the Doppler flash using a middle latitude SuperDARN Hokkaido radar and found the Doppler flash predominantly driven by changes in ionospheric refractive index.

Prior investigations have suggested that SID‐driven frequency and phase anomalies are caused by a sudden change in the traveling wave’s phase path length. Kikuchi et al. (1986) postulated two possible processes that contribute to the change in phase path length: (a) change in refractive index, which defines the EM wave speed through the media, due to enhanced plasma density in the non‐deviative layers of the ionosphere, that is, the D and lower E‐regions; and (b) change in the F‐region ray reflection height. They further postulated that the processes are related to two different geophysical phenomena. The change in refractive index is associated more with the change in photoionization in the lower ionosphere following a solar flare. While, the F‐region ray reflection height change is predominantly driven by traveling ionospheric disturbances or a decreased zonal electric field associated with a geomagnetic storm. Watanabe and Nishitani (2013) showed that the Doppler flash is predominantly due to changed ionospheric refractive index, but they were unable to conclusively comment on the location of the ionosphere responsible for the Doppler flash, that is, the D and lower E‐regions or F‐region.

Recently Chakraborty, Qian, et al. (2021) conducted a first principles‐based modeling framework study to estimate Doppler frequency shift due to change in refractive index through the whole raypath including the D, E, and F‐regions. The Doppler flash was broken down to percentage contributions from two sources and three ionospheric layers. The findings were: (a) the F‐region is the dominant contributor to the solar flare‐driven Doppler flash, and (b) change in refractive index is more important than lowering of the F‐region reflection height, which is most likely driven by the reduction in the zonal electric field.

The primary goal of this study is to understand how solar flare characteristics, geophysical conditions, and ray characteristics impact the Doppler flash. In addition, we further investigate the physical mechanisms that drive the lowering of the F‐region reflection point during solar flares. To achieve these goals, we study multiple solar flare events using SuperDARN data, JULIA radar (located at Jicamarca radio observatory) data, and the first principles‐based modeling framework described in Chakraborty, Qian, et al. (2021). The paper is organized as follows: Section 2 provides a brief introduction of the instruments and data sets used in this study; Section 3 provides an overview of the different model components of the framework; Section 4 presents statistical results; Section 5 discusses the observations and model results in the context of previous studies.

## 2 Instrumentation and Data Sets

The data sets utilized in this study were obtained from SuperDARN HF radar and Jicamarca JULIA radar. SuperDARN HF radars will provide the Doppler flash observation, while JULIA radar provides measurement of the equatorial vertical‐ion drift. This section briefly reviews these instruments and describes data from these instruments for subsequent analysis.

**Figure 1:**
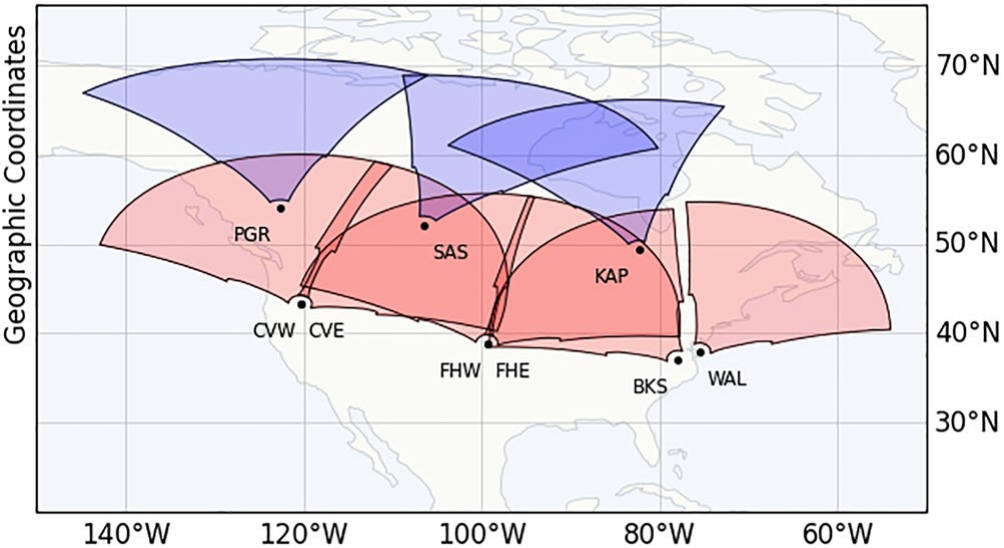
Fields‐of‐View (FoV) of Super Dual Auroral Radar Network (SuperDARN) HF radars used in this study. The FoVs of SuperDARN radars located at middle and high latitudes across the North American sectors are colored in red and blue, respectively.

SuperDARN is an international HF Doppler radar network, operating between 8 and 18 MHz, distributed in both hemispheres across middle, high, and polar latitudes. Each radar measures the line‐of‐sight (LOS) component of $\vec{E}\times \vec{B}$ drift velocity of ionospheric plasma irregularities. The field‐of‐view (FoV) of a radar typically comprises 16–24 azimuth beams, each having ∼100 range gates spaced 45 km apart beginning at 180 km. A typical integration time for each beam sounding is 3‐s or 6‐s, which results in a full radar sweep in one or 2 min, respectively. Figure 1 shows the location of the radars and their corresponding fields‐of‐views (FoVs), while Table 1 lists their geographic and magnetic locations used in this study.

**Table 1:** List of Radars Used for Statistical Study

Radar station	Station code	Geographic	Geomagnetic
Wallops Island	WAL	(37.93°, −75.47°)	(48.47°, 1.77°)
Blackstone	BKS	(37.10°, −77.95°)	(47.97°, −1.67°)
Fort Hays East	FHE	(38.86°, −99.39°)	(49.16°, −30.75°)
Fort Hays West	FHW	(38.86°, −99.39°)	(49.16°, −30.75°)
Christmas Valley East	CVE	(43.27°, −120.36°)	(49.94°, −56.80°)
Christmas Valley West	CVW	(43.27°, −120.36°)	(49.94°, −56.80°)
Kapuskasing	KAP	(49.39°, −82.32°)	(59.69°, −7.20°)
Saskatoon	SAS	(52.16°, −106.53°)	(60.85°, −41.95°)
Prince George	PGR	(53.98°, −122.59°)	(59.81°, −62.33°)

**Figure 2:**
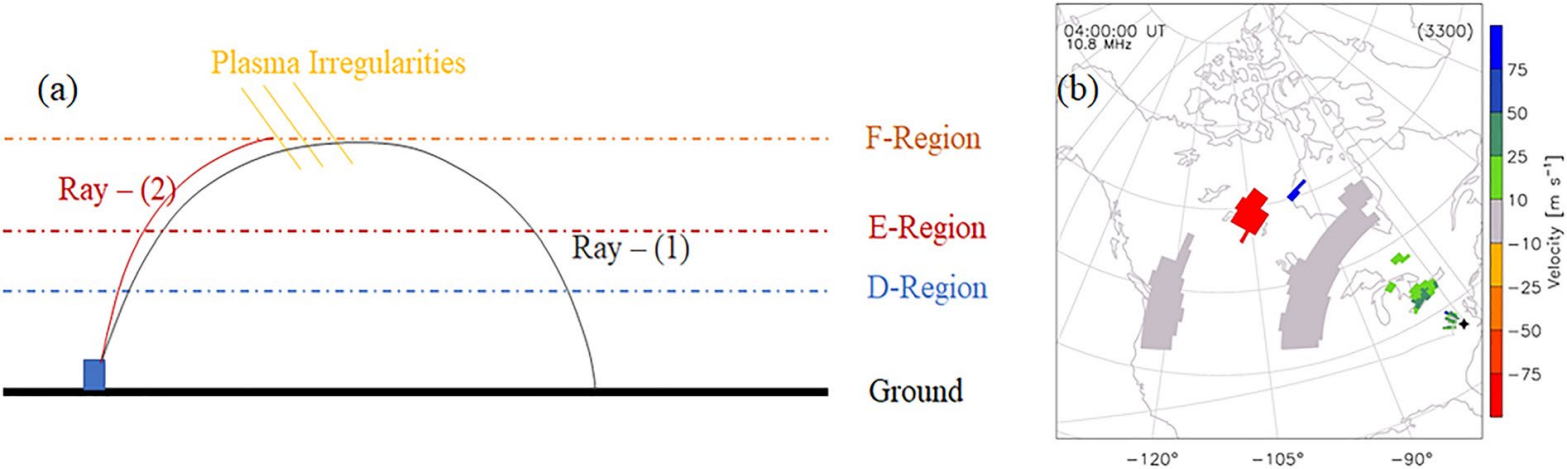
(a) Schematic plot of Super Dual Auroral Radar Network (SuperDARN) propagation modes showing ray paths of ground scatter and ionospheric scatter and (b) SuperDARN field‐of‐view scan plot, showing line‐of‐sight Doppler velocity measured by the Blackstone radar on 17 March 2015 at 4:00 UT. Velocity in panel (b) is color‐coded according to the scale on the right and ground scatter is marked gray.

SuperDARN observations primarily consist of two types of backscatter, namely, ionospheric scatter and ground scatter. Ionospheric scatter is generated when a transmitted signal is reflected from ionospheric irregularities. In the case of ground scatter, due to a high daytime vertical gradient in ionospheric refractive index, the rays bend toward the ground and are reflected from surface roughness and return to the radar following the same paths. The propagation modes illustrated in Figure 1a are called the $\frac{1}{2}$‐hop ionospheric and 1‐hop ground scatter modes. Due to the high daytime vertical refractive index gradient, ground scatter (corresponding to Ray [1] in Figure 2a) is due to the rays bent toward the ground and reflected from surface roughness and returning to the radar in the same direction. As a result, a one‐hop ground‐to‐ground communication link passes four times through the D‐region. In the ionosphere, scattering (corresponding to Ray [2] in Figure 2a) is caused by irregularities in the ionosphere that reflect the transmitted signal. In general, ground and ionospheric scattering is characterized by lower and higher Doppler velocities and narrower and wider spectral widths, respectively. Figure 2b is a SuperDARN Doppler velocity scan plot from the Blackstone radar displaying ground scatter (in gray) and ionospheric scatter (color coding based on Doppler velocity). A primary data product of SuperDARN radars is the Doppler LOS velocity of the backscatter signal, which is determined by the Doppler shift in the frequency of the backscattered signal. An increased backscattered signal frequency or blueshift indicates apparent movement toward the radar and can be identified as positive Doppler velocity. Reduced backscattered signal frequency or redshift indicates apparent movement away from the radar and is identified as a negative Doppler velocity. On most days during the day, SuperDARN observations consist of a band of ground scatter that extends over several hundred kilometers. We will only use ground scatter observations in this study since the effects of solar flares can be readily identified as a sudden bite‐out of the ground scatter band during daytime hours.

We obtained LOS ion drift velocity measurements from the Jicamarca Unattended Long‐term Investigations of the Ionosphere and Atmosphere (JULIA) radar at Jicamarca, Peru (JRO [11.95°S, 76.87°W]), which is located near the magnetic equator. The system is designed to observe equatorial plasma irregularities for extended periods and is suitable for studying day‐to‐day and long‐term variability and sudden changes of equatorial ion drift velocity in response to transients, for example, solar flares. JULIA observes backscatter from irregularities in the equatorial electrojet and generates data on vertical and zonal plasma drifts from 150 km irregularities, and nighttime spread‐F. In this study, we use average vertical ion drift observations from 150 km echoes.

## 3 Models

This section describes the first principles‐based modeling framework first reported by Chakraborty, Qian, et al. (2021), which is used here to numerically predict the LOS Doppler velocity observed by SuperDARN radars following a number of X and M‐class solar flares. We present an example output from this modeling framework for a solar flare event and compare with observations.

**Figure 3:**
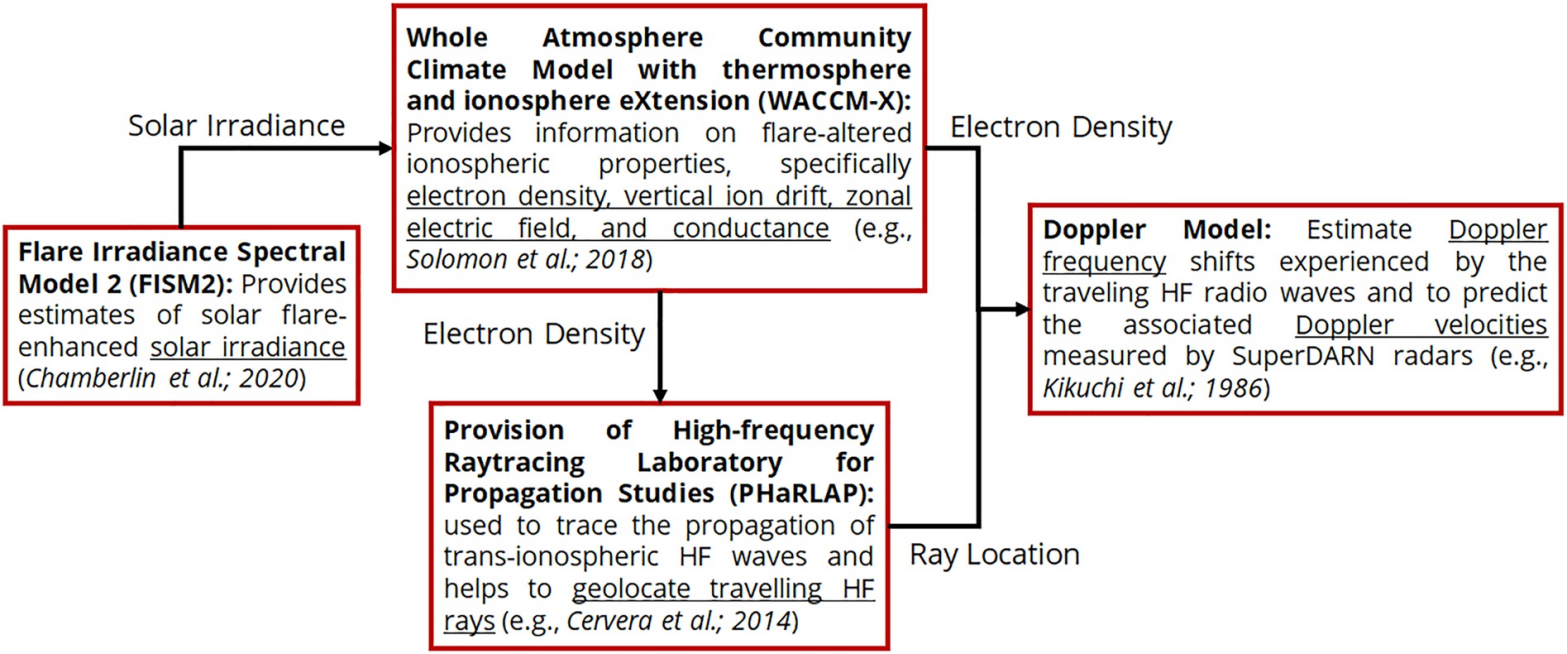
Model framework architecture for geolocating high frequency rays and Doppler effects due to solar flares showing the component modules and their interconnection.

Following Chakraborty, Qian, et al. (2021), we used a combination of four models in this framework, namely FISM2 (Flare Irradiance Spectral Model) (Chamberlin et al., 2020), WACCM‐X (Whole Atmosphere Community Climate Model with thermosphere and ionosphere eXtension) (Garcia et al., 2007; Marsh et al., 2013; Neale et al., 2013; Qian et al., 2018; Solomon et al., 2018), PHaRLAP (Provision of High‐frequency Raytracing Laboratory for Propagation Studies) (Cervera & Harris, 2014) raytracing, and Kikuchi’s Doppler model (Kikuchi et al., 1986), to predict Doppler flash phenomena observed by SuperDARN HF radars. FISM2 provides estimates of solar flare‐enhanced solar irradiance, which is used as an input to the WACCM‐X model that provides information on flare‐altered ionospheric properties, specifically electron density, vertical ion drift, zonal electric field, and conductance. We used the latest version FISM2 (Chamberlin et al., 2020) to provide solar flare spectral irradiance for the flares listed in Table 2 in this study. The PHaRLAP raytracing model is used to trace the propagation of trans‐ionospheric HF waves. Finally, we apply the Doppler theory described by Kikuchi et al. (1986), to estimate Doppler frequency shifts experienced by the traveling HF radio waves and to predict the associated velocities measured by SuperDARN radars following solar flares. Figure 3 presents a schematic of the architecture of the model framework showing how the different modules are interconnected.

**Table 2:** List of Solar Flare Events Used in This Study

Event time, UT	Flare class	Location on the solar disk	Radars affected
2015‐05‐05 22:11	X2.7	12°N 70°E, Limb	BKS, FHE, FHW, CVE, CVW, KAP, SAS, PGR
2015‐03‐11 16:22	X2.2	16°S 26°E, Disk	BKS, FHE, FHW, CVE, CVW, KAP
2015‐09‐28 14:58	M7.6	20°S 16°W, Disk	BKS, FHE, FHW, CVE, CVW, KAP, SAS, PGR
2016‐04‐18 00:29	M6.7	10°N 52°W, Disk	CVW, PGR
2014‐02‐25 00:49	X4.9	15°S 86°E, Limb	CVW
2014‐06‐10 11:42	X2.2	18°S 71°E, Limb	BKS, FHE, FHW

### 3.1 Event Study: 5 May 2015

On 5 May 2015, an X2.7‐class solar flare erupted on the Sun. At Earth, enhanced X‐ray fluxes measured by the GOES‐15 spacecraft show that the flare started, reached its peak, and ended at 22:05 UT, 22:11 UT, and 22:25 UT, respectively. HF radars observed radio blackouts for 30–60 min. Based on the maximum *K*_p_ value of 2^+^, on 5 May 2015 between 00 and 24 UT we conclude that the ionosphere was not perturbed by geomagnetic storm activity and thus this time period is suitable for observing the flare effects. We have chosen this event as a typical example in our case study paper (Chakraborty, Qian, et al., 2021) and use it here to demonstrate a data‐model comparison.

**Figure 4:**
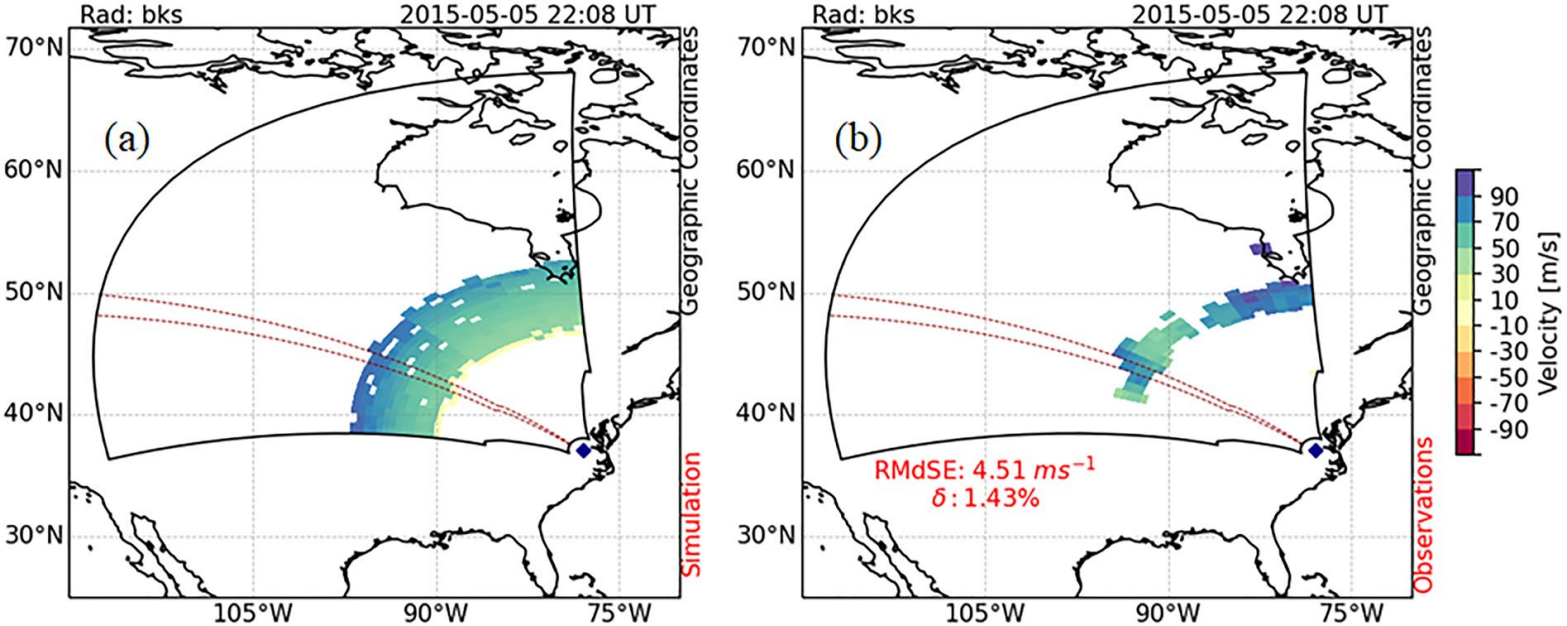
Data‐model comparison for Super Dual Auroral Radar Network Blackstone radar measurements during solar flare event on 5 May 2015: Fields‐of‐View (FoV) scan plots showing (a) Doppler velocity simulated using the model framework and (b) observations from the Blackstone radar at the peak of the Doppler flash (22:08 UT) (Adopted from Chakraborty, Qian, et al., 2021). Doppler velocity in FoV plots in both panels is color‐coded according to the scale on right. The region enclosed by the red dashed lines represents beam 7.

Figure 4 presents an example data‐model comparison during this solar flare event on 5 May 2015. Panel a shows Doppler velocity estimated using the framework while panel b shows observations from the Blackstone radar at the peak of the Doppler flash (5 May 2015, 22:08 UT). Velocity is color‐coded by the color bar on the right. Both panels show scatter extending over an annular region corresponding to returns from the Earth surface after reflection from the ionosphere. The scatter is associated with a velocity measurement that is due to the change in phase path length of the ray. Both model output and observations record a surge in Doppler velocity up to 100 ms^−1^. Note that the radar observations suffered from the initial HF absorption phase of the SWF; thus, the radar did not receive backscattered echoes for all the range‐cells uniformly. The region enclosed by the red dashed line in panels a, b represents beam 7. To compare the modeled output against the observations, we have used two different metrics, root‐median‐squared‐error (RMdSE) and mean percentage error (MPE, *δ*). To estimate RMdSE and MPE we only consider range‐cells with valid observations and discard all range cells with no observations. The RMdSE and MPE for this case are provided in panel b. The framework is able to predict observations during the peak of the Doppler flash (∼22:08 UT) with an RMdSE of 4.51 ms^−1^ and a MPE (*δ*) of 1.43%, which suggests a good agreement between the data and model output.

**Figure 5:**
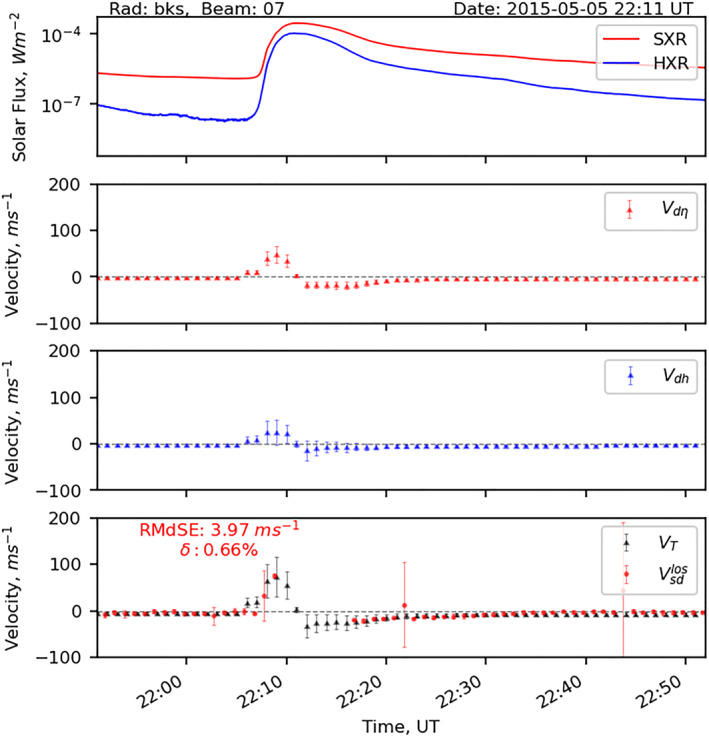
Time‐series plot (a) from GOES‐15 X‐ray sensor data, and plot along beam 7 of the Blackstone radar (b) modeled Doppler velocity due to change in refractive index, (c) modeled Doppler velocity due to change in ray reflection height, and (d) total Doppler velocity from the model (in gray) and observation (in red) with outliers are being characterized by the vertical red error bars. Root‐median‐squared‐error and MPE (*δ*) between modeled and observed Doppler velocity is provided in panel (d). (Adopted from Chakraborty, Qian, et al., 2021).

Figure 5 presents a data‐model comparison from beam 7 of the SuperDARN Blackstone radar observations for the 1‐hr time interval between 21:51–22:52 UT. Panel a present shows soft X‐ray (SXR, in red) and hard X‐ray (HXR, in blue) solar flux data from the GOES‐15 satellite. Panels b, c present modeled Doppler velocity contributed by the change in refractive index and change in the ray reflection height, respectively. The bottom panel d presents the total Doppler velocity estimated using the model overlaid with the observations from the radar. Error bars in all panels represent variations of Doppler velocity along beam 7. Due to the HF absorption effect radar observations are missing for ∼7 min, 22:10–22:17 UT. Figure 5d shows that the SWF phenomenon lasted for ∼17‐min. The radio blackout event was preceded by a sudden enhancement of apparent Doppler velocity or a blue shift at 22:08 UT. Hence we do not consider this period while estimating RMdSE and MPE. The analysis indicates that the model is able to replicate velocity observations prior to the flare, at the peak of the Doppler flash, and post‐flare with an RMdSE of 3.72 ms^−1^ and an MPE of 0.67%. The flare‐enhanced electron densities at D, E, and F‐region altitudes produce a sudden change in the refractive index and a reduction in the eastward electric field. The change in refractive index alters the phase path length of the traveling radio wave which manifests as a Doppler blue the SuperDARN observations (Chakraborty, Qian, et al., 2021). Additionally, the flare‐driven reduction in the eastward electric field lessens the vertical $\vec{E}\times \vec{B}$ drift. This lowers the height of the peak electron density of the F2 region, which helps to refract (reflect) HF rays to the ground from relatively lower altitudes and creates additional Doppler blue shift in the SuperDARN observations (Chakraborty, Qian, et al., 2021). Both of these sources cumulatively create a blue shift in the SuperDARN observation. Chakraborty, Qian, et al. (2021) also suggested that the Doppler effect is directly proportional to the rate of change in electron density, which is proportional to the rate of change in solar irradiance. Here the peak of the observed and modeled Doppler flash occurred at 22:08 UT as shown in panels b–d. This timing coincides with rising phase of the solar flux. In addition, at the peak of solar flare (at 22:10 UT), we observe a drop in Doppler flash that is consistent in both observations and model.

## 4 Results

This section presents statistics from six flare‐driven Doppler flash events, listed in Table 2, modeled using the framework described in Section 3. We compare results against our previous study and describe the probable reasons for their similarities or differences. Next, we conduct an event study to compare the modeled vertical ion drift with observations from JULIA radar during an X17 class flare. We investigate the possible reason behind the change in vertical ion drift and dayside zonal electric field following extreme solar flares.

### 4.1 Statistical Study

To characterize the dominant ionospheric drivers of the Doppler flash, we have conducted a statistical study using the simulated results for six solar flare events listed in Table 2, which presents flare peak time, class, location on the solar disk, and flare affected SuperDARN radar codes. Here we compare the statistics obtained from multiple radar events against the findings reported in Chakraborty, Qian, et al. (2021).

**Figure 6:**
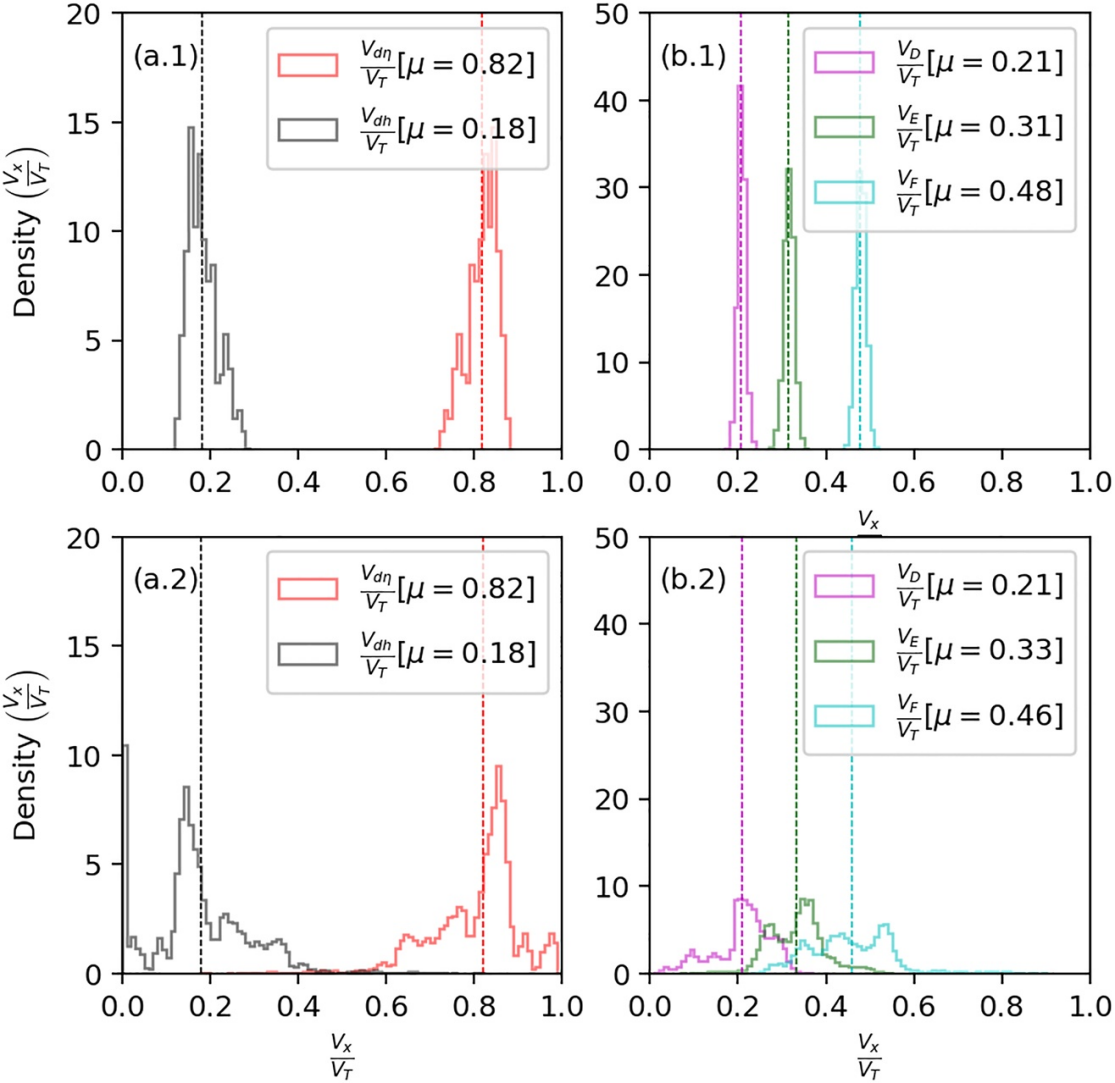
Histograms of (a.1, a.2) percentage of Doppler velocity contributed by the change in refractive index (in red) and change in ray reflection height (in black), (b.1, b.2) percentage of Doppler velocity contributed by the D, E, and F‐regions in magenta, green, and cyan. Mean (*μ*) for each population is provided in the legend. The top and bottom rows present statistics against one solar flare event (adopted from Chakraborty, Qian, et al., 2021) and multiple solar flare events (listed in Table 2), respectively.

Figure 6 presents the comparison between the histograms of percentage modeled Doppler velocity contributed by two ionospheric drivers (changes in refractive index and ray reflection height) and three layers (D, E, and F). The top and bottom rows show statistics against one solar flare event (adapted from Chakraborty, Qian, et al., 2021) and multiple solar flare events (listed in Table 2), respectively. Against each radar event, we simulated data for all beams (0–23), with different elevation angles (15°–35°), and during the whole period of Doppler flash observed starting ∼3 min before the peak and lasting ∼3–4 min. This amounts to ∼35,000 simulated data points. Panels a.1, a.2 present histograms of relative contributions of the Doppler flash due to the change in refractive index (in red) and the change in ray reflection height (in black). Panels b.1, b.2 present histograms of relative contributions of the Doppler flash by the D, E, and F‐regions in magenta, green, and cyan, respectively. Colored vertical dashed lines in all the panels represent the population means (*μ*).

Through statistical analysis of one event presented in Chakraborty, Qian, et al. (2021), we demonstrated that, on average, (a) relative contributions to the Doppler flash from the change in refractive index and change in the ray reflection height are ∼82% and ∼18%, respectively; and (b) relative contributions of the D, E and F‐regions are ∼21%, ∼31%, and ∼48%, respectively. In this statistical study, we find that the mean statistics did not change much, that is, on average, (a) relative contributions to the Doppler flash from the change in refractive index and change in the ray reflection height are ∼82% and ∼18%, respectively; and (b) relative contributions of D, E and F‐regions are ∼21%, ∼33%, and ∼46%, respectively. However, we do see significantly more variance around the mean statistics (*μ*) in the bottom row than in the top row, for example, density curves presented in panel b.2 overlap on top of each other. We propose the following geophysical and radar parameters contribute to these variations: flare location on solar disk, solar zenith angle (SZA), radar operating frequency, and ray elevation angle. In the following paragraphs, we investigate whether contributions from the D, E and F‐regions have a significant dependence on these factors.

**Figure 7:**
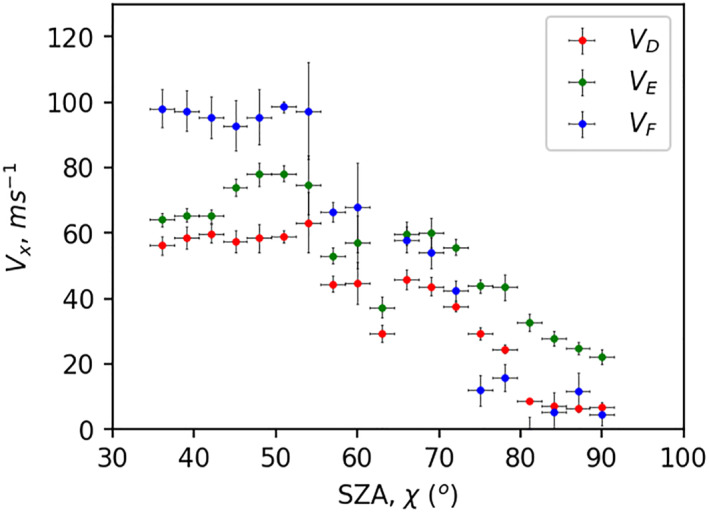
Variations of modeled Doppler velocity contributed by the D, E, and F‐regions, in red, green, and blue, with solar zenith angle (SZA). Dots represent median velocity within 3° SZA bin, represented by horizontal lines, and vertical error bars represent median absolute deviation within 3° SZA bin.

Figure 7 presents variations in the total contribution to modeled Doppler flash by the D (*V*_D_), E (*V*_E_), and F (*V*_F_) regions with SZA. Each dot represents the median velocity within a 3° wide SZA bin and median‐absolute‐deviation (median absolute deviation [MAD]) are shown by vertical error bars. We see at lower SZA (≤55°) *V*_F_ is the highest contributor to the Doppler flash, while at higher SZA (≥60°) contributions from the D and E‐regions become more significant. Flare enhanced X‐rays are responsible for D and lower E‐region photoionization, while EUV is the dominant source of ionization in the upper E and F‐region (Qian et al., 2010). At larger SZAs, solar ionizing radiation must penetrate a thicker atmosphere, leading to less ionization and a higher F2 peak. Figure 7 shows that this bias favors Doppler flash contribution by the D and E‐regions over the F‐region at larger SZAs and creates a variation in percentage Doppler velocity contribution from different ionospheric layers with SZA.

**Figure 8:**
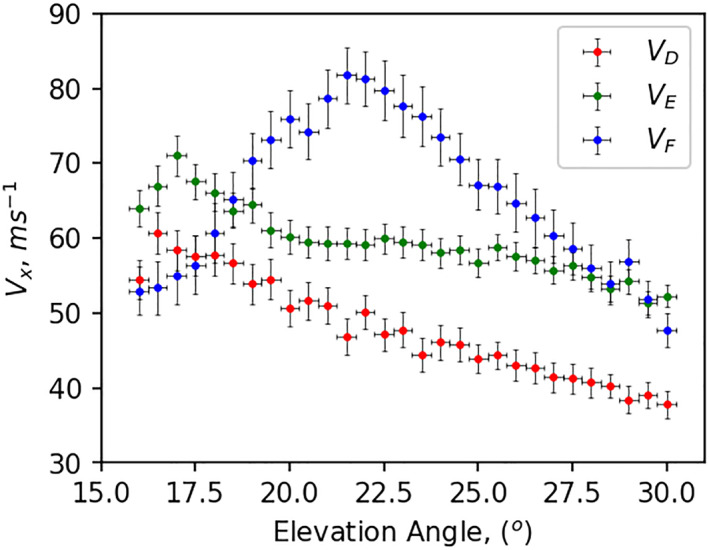
Variations of modeled Doppler velocity contributed by the D, E, and F‐regions, in red, green, and blue, with elevation angle. Dots represent median velocity within 1° elevation angle bin, represented by horizontal lines, and vertical error bars represent median absolute deviation within 1° elevation angle bin.

Elevation angle also plays a crucial role in the propagation path through the various regions of the ionosphere. Figure 8 presents variations in the total contribution to modeled Doppler flash by the D, E, and F regions versus ray elevation angle (*α*). Dots represented here are the median velocity within each 1° elevation angle bin and MAD are shown by vertical error bars. Doppler velocity contributed by the F‐region is the smallest at lower elevation angles (∼16°), peaks at moderate elevation angles (∼22°), and drops below that of the E‐region’s at higher elevation angles (∼30°). Contributions from the D and E‐regions also showed similar behavior with elevation angles but peak at different elevation angles, ∼16° and ∼17°, respectively. From Equation 1 in Chakraborty, Qian, et al. (2021), we infer the change in Doppler frequency is inversely and directly proportional to the cosine of elevation angle and path length (d) through non‐deviative ionosphere (D and lower E‐regions), respectively, that is, $\Delta f\propto \frac{d}{\cos\;\alpha }$. Note that the path length through the non‐deviative ionosphere decreases with elevation angle. Rays with higher elevation angles penetrate deeper into the F‐region, while rays with lower elevation angles traverse much longer distances in the D and lower E‐regions. Thus, contributions from the F‐region increase with elevation angle. On the other hand, for the rays with a higher elevation angle, the path length (d) through the non‐deviative ionosphere decreases, which again causes a reduction in Doppler velocity contribution from the F‐region. Thus, taking these two factors together, we see an effective peak in Doppler velocity contributed by the F‐region at ∼22° and gradual drop on either side.

**Figure 9:**
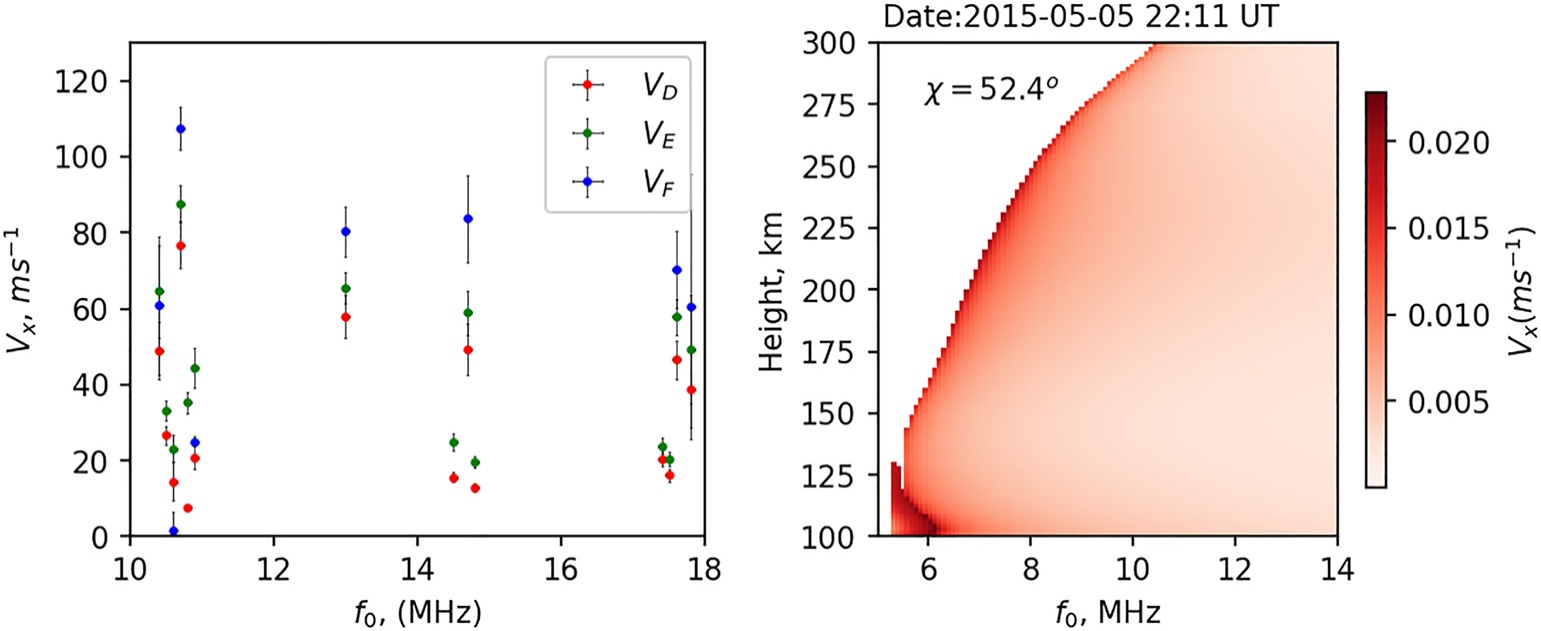
(a) Variations of modeled Doppler velocity contributed by the D, E, and F‐regions, in red, green, and blue, with radar operating frequency, (b) altitude profile of Doppler velocity (*V*_*x*_) with radar operating frequency during a flare event on 5 May 2015 at 22:11 UT. Change in refractive index is estimated for vertical propagation.

Radar operating frequency plays a significant role in controlling the propagation path and reflection height. Figure 9a presents the variation in the total contribution to Doppler flash by the D, E, and F regions versus radar operating frequency. Dots represented here are the median velocity within each 1 MHz frequency bin, and MAD are shown by vertical error bars. As radars are primarily operated on pre‐programmed frequencies, there is no continuous coverage in radar operating frequency. From Figure 9a, we do not see any clear relationship between the contributions from different ionospheric layers and radar operating frequency. We conducted a near‐vertical propagation (NVIS) simulation study at different radar frequencies to show the Doppler velocity variations with height. Figure 9b presents the Doppler velocity distribution with height during a solar flare on 5 May 2015 from the NVIS simulation study. As a NVIS propagating ray is not able to pass the corresponding ionospheric plasma frequency, the Doppler velocities for each frequency bin along the path can only be estimated up to the reflection height. It can be seen that the Doppler velocity maximizes at the ray reflection point. As higher frequencies penetrate deeper into the F‐region altitudes, we see an increase in *V*_F_ and decrease in *V*_D_ with radar operating frequency.

**Figure 10:**
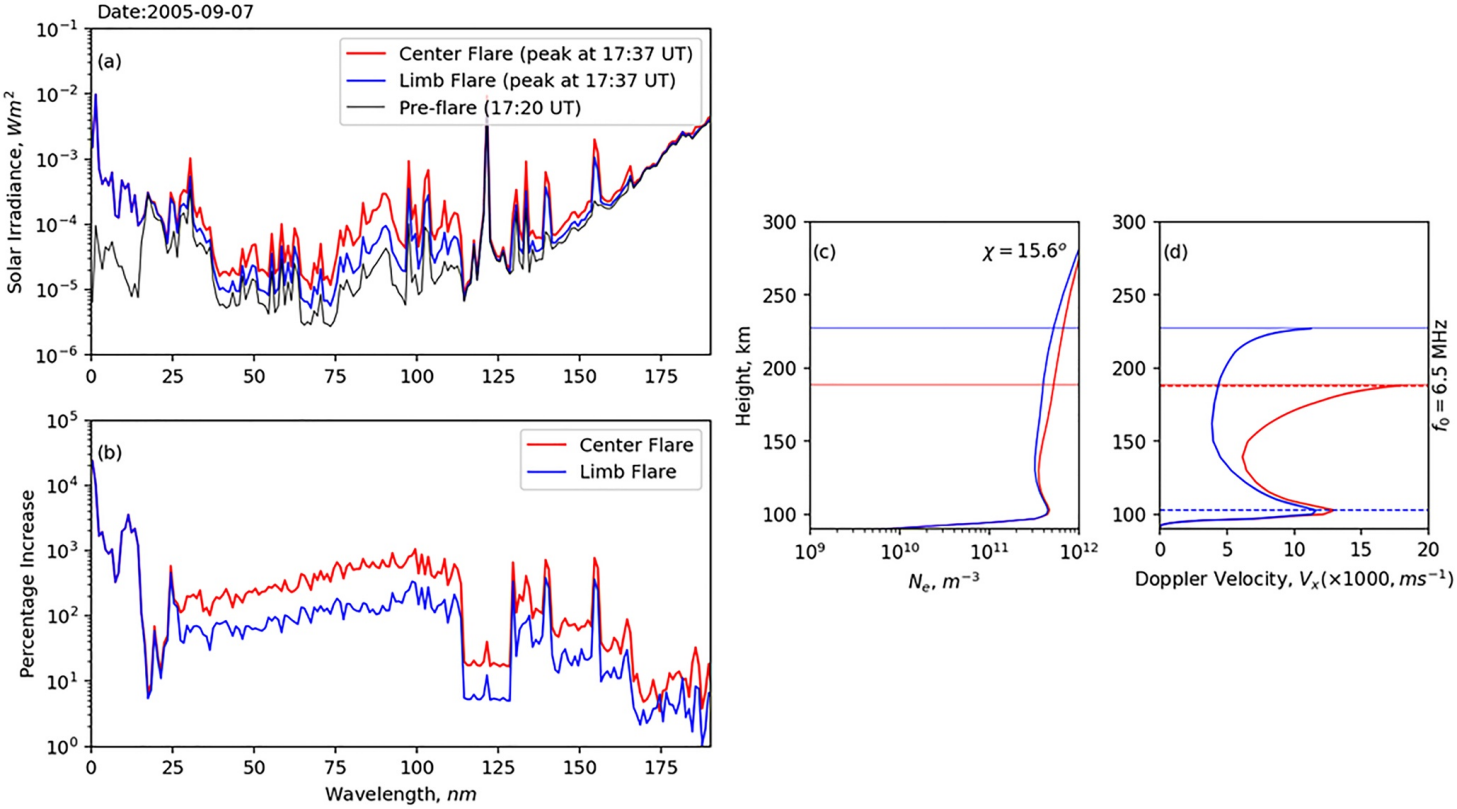
FISM2 solar irradiance spectrum from 0.05 to 195 nm for two identical X17 disk and limb solar flares on 7 September 2003 and their effects on the ionosphere at solar zenith angle 15.6°: (a) Solar spectra before (in black), at peak for center flare (in red), and at peak for limb flare (in blue); (b) percentage increase in solar irradiance to the pre‐flare conditions; (c) height distribution of modeled electron density for disk (in red) and limb (in blue) flares; and (d) height distribution of Doppler velocity (*V*_*x*_) estimated for 6.5 MHz NVIS propagation. Solid horizontal lines in panels (c–d) represent height of the critical frequency for 6.5 MHz, while dashed line in panel (d) represent height of maximum Doppler velocity. Solid horizontal lines in panels (c–d) represent height of the critical frequency for 6.5 MHz, while dashed line in panel (d) represent height of maximum Doppler velocity.

Based on the location on the solar disk, flares can be categorized as disk or limb. The disk flares are typically within ∼50° from disk center, while limb flares are located beyond 50° from the solar disk (Ackermann et al., 2017). Qian et al. (2010) showed the significance of center‐to‐limb variations (CLVs) on the ionospheric flare effects using two simulated X17 flares, which mimic properties of the X17 solar flare that occurred on 7 September 2005. These two simulated flares were identical except that they originated at different locations on the solar disk. One occurred near the disk center (∼1°), and the other occurred near the limb (∼85°). Figure 10 presents both flare spectra and their impacts on the Doppler flash. The flares started at 1,720 UT, and soft X‐rays peaked at 17:37 UT. Figures 10a and 10b present two flare spectra compared against one background spectrum (in black) and percentage changes of solar irradiance, respectively. In the short wavelength ranges (≤16 nm, responsible for ionizing the D‐region), there was no difference in the irradiance enhancement produced by the center flare and the limb flare; between 16 and 27 nm, enhancement of irradiance by the center flare was a few percent stronger compared to the limb flare. Such differences became more significant for wavelengths longer than 27 nm, which dominate ionization in the E‐ and F‐region. We see ∼50%–250% enhancement in irradiance spectrum produced by the center flare than the limb flare in the EUV range (27–105 nm). Figures 10c and 10d show height distributions of electron density and Doppler velocity for a 6.5 MHz NVIS propagation, respectively, for two simulated flares near the subsolar point (SZA, chi ∼ 16°). Solid horizontal lines represent reflection points, while dashed lines in panel d represent the height of maximum Doppler velocity. Note that, for the limb flare, the peak in Doppler velocity is in the lower E‐region (∼110 km), while for the disk flare it is in the F‐region (∼185 km). This indicates that the flare location affects Doppler velocity contribution from different ionospheric layers.

### 4.2 Flare‐Driven Change in Ray Reflection Height, Vertical Ion‐Drift, and Ionospheric Conductivity

Chakraborty, Qian, et al. (2021) described that the change in reflection height might also play a role in the Doppler flash observed by SuperDARN radars, and the change in ray reflection height was due to reduction in vertical plasma drift. The statistical results presented here show that the change in reflection height is a secondary contributor to the Doppler flash. This subsection examines the reduction in vertical ion‐drift using a data‐model comparison during an X17 solar flare on 7 September 2005.

**Figure 11:**
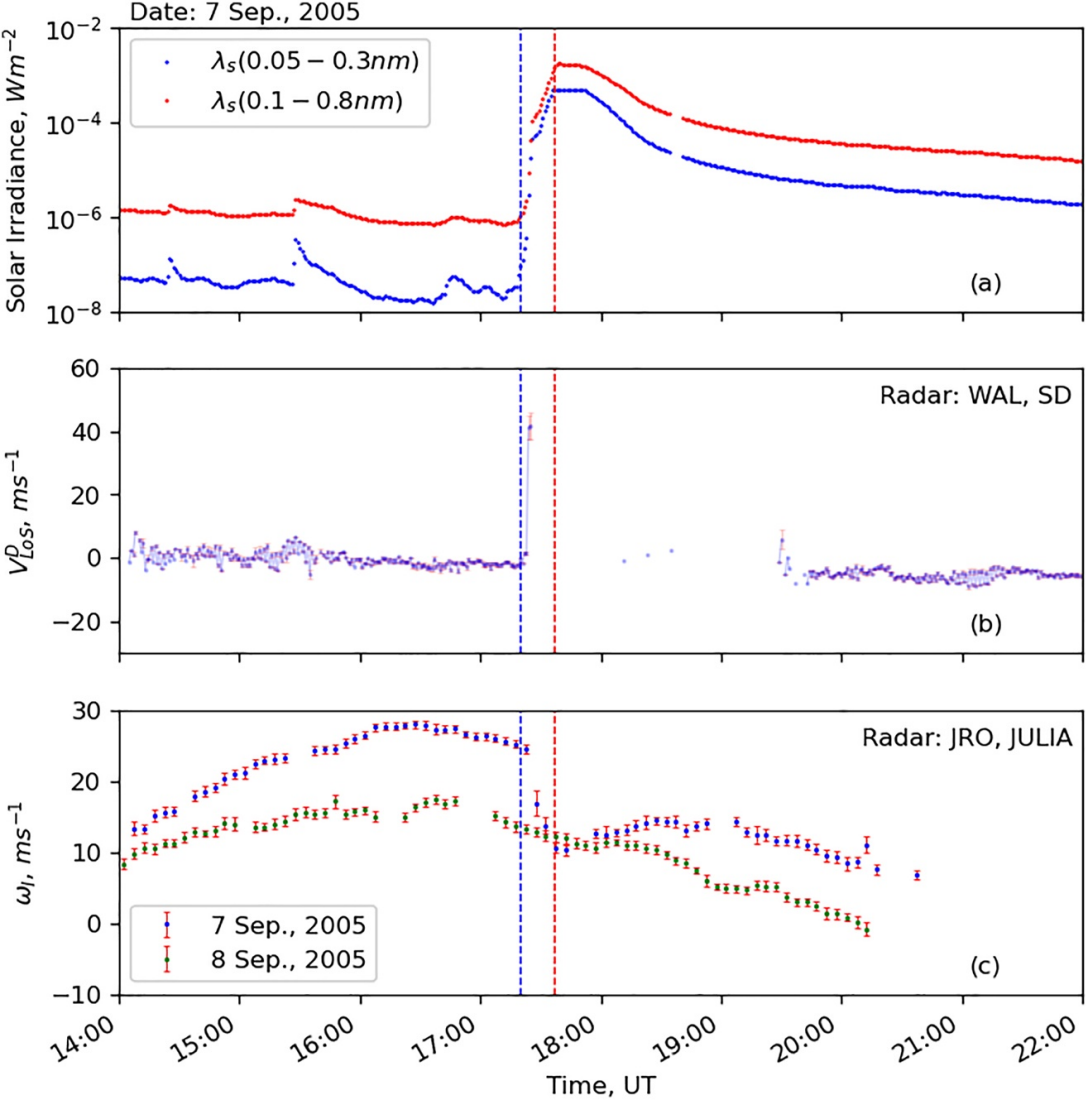
GOES‐15 satellite, Super Dual Auroral Radar Network (SuperDARN) Blackstone radar, and ISR Jicamarca radar measurements during an X17 solar flare event on 7 September 2005: (a) GOES‐15 X‐ray flux in SRX (in red) and HRX (in blue) waveband, (b) Doppler line‐of‐sight velocity from SuperDARN Wallops island radar color‐coded by the color bar on the right, (c) vertical ion drift from Jicamarca JULIA instrument, on 7 September (in blue) and 8 September (in green) for reference. Outliers are characterized by the large uncertainty values indicated by the vertical red lines.

Figures 11a–11c present the solar flare details from the GOES‐15 X‐ray flux sensor and its effects recorded in observations from the SuperDARN Wallops Island (WAL) and Jicamacra JULIA instrument, respectively. Specifically, the middle and lower panels show the temporal evolution of the Doppler flash at mid‐latitudes and the equatorial vertical ion drift, respectively. For comparison with no‐flare condition, the vertical ion‐drift observations from 8 September 2005 (green dots) are also presented in panel c. The flare started at 17:20 UT, and peaked at 17:37 UT, which are identified by vertical blue and red dotted lines. The WAL data show the mid‐latitude Doppler flash occurred at ∼17:25 UT, while the JULIA data shows the equatorial vertical plasma drifts decreased after the flare and reached a minimum value of ∼10 ms^−1^ at 17:37 UT. SuperDARN WAL radar observations are severely affected by the blackout (peak of HF absorption Chakraborty et al., 2018) between 17:25–19:30 UT, and thus the observations suffer from the bite‐out effects of the flare‐driven SWF.

**Figure 12:**
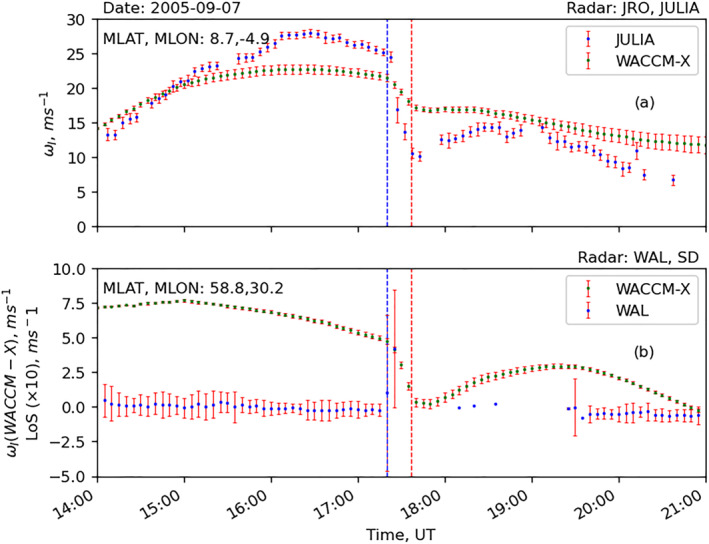
Time‐series plot of data‐model comparison between modeled vertical ion drift and radar observations during an X17 solar flare event on 7 September 2005: (a) average vertical ion drift data from WACCM‐X (in green) and Jicamarca JULIA instrument, and (b) average vertical ion drift data from WACCM‐X (in green left axis) and line‐of‐sight (LOS) Doppler velocity from Super Dual Auroral Radar Network WAL radar (in blue right axis).

Figure 12a presents the data‐model comparison between vertical plasma drift data from JULIA and the WACCM‐X model. Similarly, Figure 12b presents Doppler flash data from WAL radar and modeled vertical ion‐drift from WACCM‐X at the middle of WAL’s FoV. Magnetic coordinates of both radars are provided in the panels. The following features can be identified in the figure: (a) WACCM‐X reproduces the reduction of the observed vertical plasma drifts but underestimates the JRO observation by a factor of ∼2 at the peak of the solar flare; (b) reduction in the modeled vertical ion‐drift velocity is more severe at lower latitudes than middle‐latitudes; and (c) the Doppler flash observed in SuperDARN WAL radar coincides with the drop in vertical ion‐drift observation from JULIA and in the WACCM‐X simulation.

**Figure 13:**
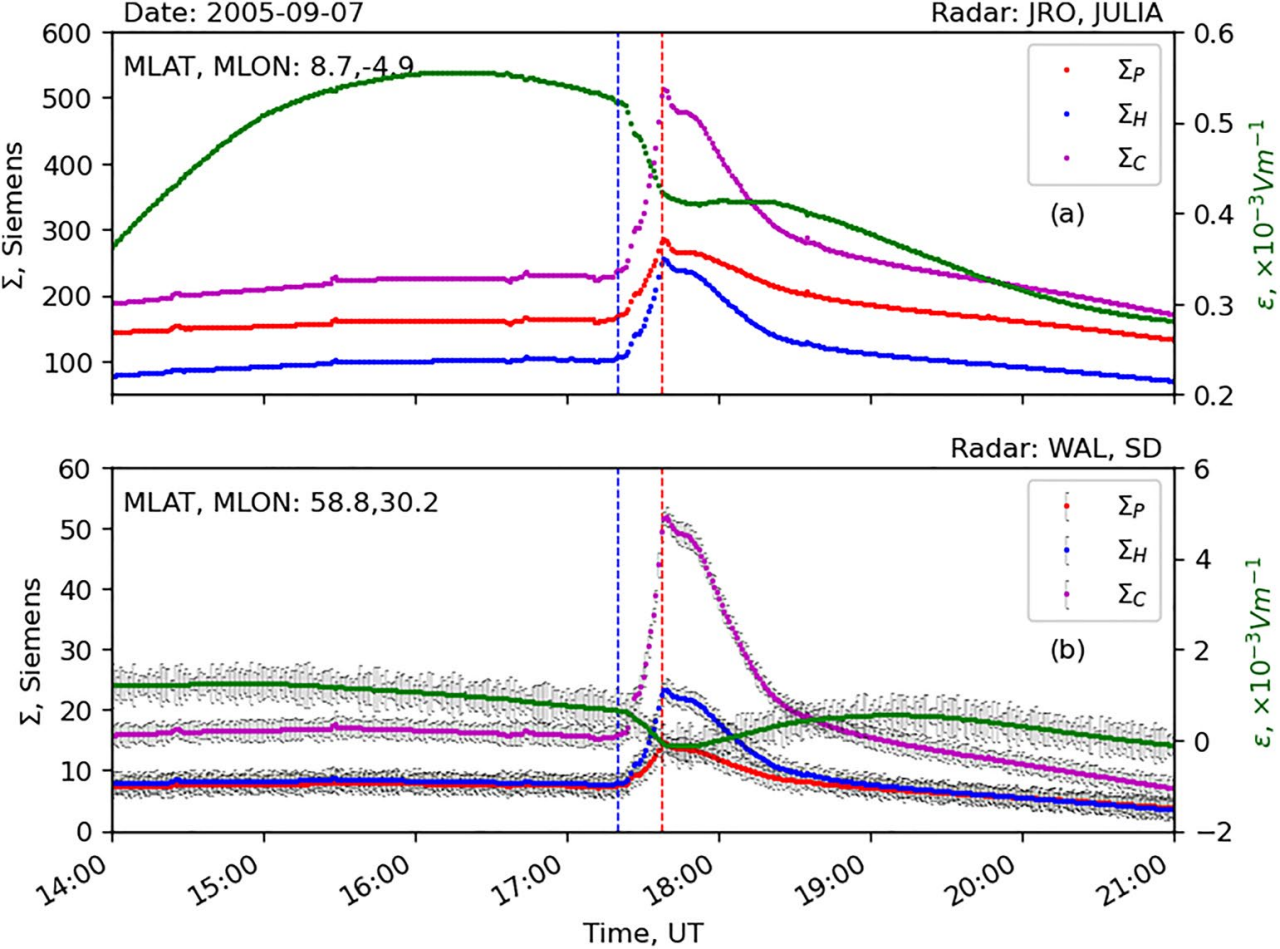
Modeled conductance (left axis) and zonal electric field (*E*_*y*_, right axis) from the WACCM‐X model. Time evolution of Pederson conductance (in red), Hall conductance (in blue), Cowling conductance (in magenta), and zonal electric field (in green) above (a) Jicamarca JULIA instrument and (b) Fields‐of‐View of the Super Dual Auroral Radar Network WAL radar.

Chakraborty, Qian, et al. (2021) suggested that the reduction in vertical plasma drift is related to the reduction in the zonal electric field (*E*_*y*_) in the sunlit side of the Earth following a solar flare. However, the study did not investigate the process that drives reduction in the zonal electric field. Here we present the modeled zonal electric field and ionospheric conductance from the WACCM‐X model to examine the source of the electric field perturbation following a solar flare. Figure 13 presents the temporal evolution of the simulated zonal electric field and Pedersen (Σ_P_, in red), Hall (Σ_H_, in blue), and Cowling ($\Sigma_{C}=\Sigma_{P}+\frac{\Sigma_{H}^{2}}{\Sigma_{P}}$, in magenta) conductance over JULIA and the WAL FoV, respectively. Vertical black lines in panel b represent the MAD across the WAL FoV. Noteworthy is a sudden increase in ionospheric conductance following the X17 flare that coincides with the drop in the zonal electric field. We know, at steady state, ionospheric currents can be assumed to be divergence‐free (Heelis, 2004; Yamazaki & Maute, 2017), $\nabla .\vec{j}=0$ with $\vec{j}=\bar{\sigma }.\vec{E}$, where: $\vec{j}$, $\vec{E}$, and $\bar{\sigma }$ are the ionospheric current density vector, electric field vector, and conductivity tensor, respectively. Large enhancement of electron density in the E region during the solar flare increases both Hall and Pedersen conductivities, and the Cowling conductance, but the Cowling conductance increases more strongly than the Pedersen and Hall conductance (Figure 13). Whereas the dynamo source current tends to scale with the Pedersen conductance, the effective conductance connecting the zonal current to the zonal electric field is the Cowling conductance Liu, Qian, et al. (2021). This results in the increase of the Cowling conductance being larger than the flare increase in the dynamo source current from the Pedersen conductance increase. In order to maintain global current continuity, the zonal electric field must weaken during the flare to prevent the Cowling closure current from increasing excessively in relation to the global dynamo source current. The weakened eastward electric field causes weakened ionospheric vertical drift during the flare.

## 5 Discussion

In this study, we have presented statistical analysis of multiple Doppler flash events using simulated data from a physics‐based ray‐tracing model framework and observations from SuperDARN HF radars. In addition, we also presented a data‐model comparison between the Jicamarca JULIA radar and WACCM‐X vertical ion‐drift to understand the dynamics and associated processes behind the flare‐driven drop in F‐region HF ray reflection height.

The average statistics (listed in Table 3) presented here show great agreement with our previous study (Table 2 of Chakraborty, Qian, et al., 2021). We showed that ray elevation angle, SZA, operating frequency, and flare location play a significant role in determining the primary contributor of the Doppler flash among the three layers of the ionosphere, which contribute to the overlap in distributions presented in Figure 2d. Rays with different frequencies and elevation angles can reach different ionospheric layers. Consequently, operating frequency and elevation angle create a spread in the distribution of relative contributions from D, E, and F‐regions to the Doppler flash. In addition, both SZA and flare location on the solar disk plays a decisive role in determining how much each ionospheric layer contributes to the Doppler flash during solar flares, which also contributes to the spread in the distribution. Location of the transmitter/receiver or SZA creates a grazing angle effect, which forces solar irradiance to pass through a thicker atmosphere for a larger SZA, which increases the atmospheric extinction coefficients for EUV wavebands and leads to lesser photoionization at F‐region altitudes. Location of a flare on the solar disk also creates CLVs that modulate the EUV, UV, and FUV wavebands of solar irradiance that reach the upper atmosphere but not the X‐rays (e.g., Worden et al., 2001). These relatively higher wavebands (optically thicker than X‐rays) undergo more absorption by the solar atmosphere for the flares originated at the solar limb than the solar disk (Chamberlin et al., 2008). Consequently, energy deposition by these wavebands that are predominantly responsible for photoionization in the upper E and F‐regions produces less ionization at those altitudes for flares originated near the solar limb than those on the solar disk.

**Table 3:** Percentage (%) Contributions by Different Ionospheric Layers and Drivers to the Doppler Flash, Modeled Using the Framework Described in Section 3

	Total contribution to Doppler flash (*μ*, *σ*)	Contribution from the change in refractive index (*μ*, *σ*)	Contribution from the change in reflection height (*μ*, *σ*)
D‐region	(21%, 6.5%)	(21%, 6.5%)	(0%, NA)
E‐region	(33%, 6.8%)	(33%, 6.8%)	(0%, NA)
F‐region	(46%, 10.2%)	(28%, 11.2%)	(18%, 11.8%)

We also showed that a solar flare changes the phase path length of the traveling radio wave by lowering the ray reflection height, which is related to a drop in vertical $\vec{E}\times \vec{B}$ plasma drift. Chakraborty, Qian, et al. (2021) suggested that flare‐driven reduction in the zonal electric field is responsible for the reduction in vertical $\vec{E}\times \vec{B}$ plasma drift motion at lower and middle latitudes. Liu, Qian, et al. (2021) showed that the change in prompt penetration electric field (PPEF) and internal dynamo process following a solar flare are due to solar flare‐induced conductance enhancements (Curto, 2020; Kikuchi, 2014; Liu, Wang, et al., 2021; Sumod & Pant, 2019). This change in flare‐driven PPEF and internal dynamo process reduces the daytime eastward electric field. A complete explanation behind the flare‐driven changes in PPEF and its impact on high and polar latitudes requires a coupled magnetospheric‐thermospheric‐ionospheric model that is beyond the scope of this study. However, the solar flare‐induced ionospheric conductance enhancement and its effects on the thermosphere/ionosphere internal dynamo process in the middle and lower latitudes is more straightforward and can be explained using the results obtained from the WACCM‐X.

Finally, we found that Hall and Pedersen conductance increase by more than a factor of ∼2 during the peak of the X17 solar flare estimated using the WACCM‐X model, which is consistent with the results found in Chen et al. (2021). Flare‐enhanced photoionization enhances Hall and Pedersen conductivity in such a way that it increases the Cowling conductance by about a factor of ∼2.5 during the peak of the flare at lower and middle latitudes. To maintain global current continuity, a reduction in the zonal electric field is needed to compensate for the enhancement in ionospheric conductance. This reduction in the zonal electric field following a flare reduces the vertical $\vec{E}\times \vec{B}$ plasma drift and fountain effects in the middle and lower latitudes (Qian et al., 2012). The reduction in vertical plasma drift leads to an enhanced density at lower altitudes and thus reduces the radio signal reflection height.

## 6 Conclusions

In this study, we conducted a statistical analysis for multiple SuperDARN radar Doppler flash events driven by multiple solar flares, using a physics‐based raytracing modeling framework developed in Chakraborty, Qian, et al. (2021). We analyzed 28 solar flare‐driven radar Doppler flash events to examine the relative contributions from the change in ionospheric refractive index and the change in ray reflection height, and the relative contributions by the D‐, E‐, and F‐ regions. We found:

1. Distributions of the relative contributions from the change in ionospheric refractive index and the change in ray reflection height are statistically different (negligible overlap), with average contributions of ∼82% and ∼18%, respectively. This is consistent with the results from our previous single‐event study of Chakraborty, Qian, et al. (2021).
2. The F and E‐regions play dominant roles in the Doppler flash. On average, they contribute ∼46% and ∼33% of the flare‐driven Doppler flash, respectively. This is again in good agreement with our previous single‐event study (Table 2 of Chakraborty, Qian, et al., 2021). However, the statistics obtained in this study show significant spread around the mean percentage contributions from the D‐, E‐, and F‐ regions.
3. Ray elevation angle, SZA, operating frequency, and flare location play significant roles in determining the primary contributor of the Doppler flash among the three layers of the ionosphere, causing the spreads from the statistical means.
4. The reduction in vertical ion‐drift is due to flare‐driven increases in Hall and Pedersen conductance, and the resultant reduction in daytime eastward zonal electric field.

Our future work will examine other SFEs, namely, magnetic crochet, enhanced equatorial electrojet, counter electrojet, and the *S*_q_ current systems, and their relationship to enhanced ionospheric conductance and the Doppler flash.

## Data Availability

We would like to acknowledge the use of computational resources (https://10.5065/D6RX99HX) at the NCAR‐Wyoming Supercomputing Center provided by the NSF and the State of Wyoming, and supported by NCAR’s Computational and Information Systems Laboratory for the WACCM‐X simulations. The Jicamarca Radio Observatory is a facility of the Instituto Geofisico del Peru operated with support from the NSF AGS‐1732209 through Cornell University. We wish to acknowledge the use of the NOAA/GOES X‐ray data (from https://satdat.ngdc.noaa.gov/sem/goes/data/) for flare confirmation, analysis, and use as model inputs. We thank all participants in the worldwide SuperDARN collaboration for the distribution of SuperDARN data via http://vt.superdarn.org/tiki-index.php?page=Data+Access. Download the data used in this study via following link http://vt.superdarn.org/tiki-index.php?page=Examine%20Fit%20Contents. The authors acknowledge Advanced Research Computing at Virginia Tech for providing computational resources and technical support that have contributed to the results reported within this paper (https://198.82.212.30). The majority of analysis and visualization was completed with the help of free, open‐source software tools such as matplotlib (Hunter, 2007), IPython (Perez & Granger, 2007), pandas (McKinney, 2010), PyForecastTools (Morley, 2018), and others (e.g., Millman & Aivazis, 2011). Our code is published in GitHub repository (Chakraborty, 2022).
